# Bedside clinical data subphenotypes of critically ill COVID-19 patients: a cohort study

**DOI:** 10.5935/0103-507X.20210027

**Published:** 2021

**Authors:** Raul dos Reis Ururahy, César Albuquerque Gallo, Bruno Adler Maccagnan Pinheiro Besen, Marcelo Ticianelli de Carvalho, José Mauro Ribeiro, Rogério Zigaib, Pedro Vitale Mendes, Marcelo Park

**Affiliations:** 1 Internal Medicine Department, Hospital das Clínicas, Faculdade de Medicina, Universidade de São Paulo - São Paulo (SP), Brazil.; 2 DEmergency Department, Intensive Care Unit, Hospital das Clínicas, Faculdade de Medicina, Universidade de São Paulo - São Paulo (SP), Brazil.

**Keywords:** COVID-19, SARS-CoV-2, Cluster analysis, Algorithms, Phenotypes, Intensive care units, COVID-19, SARS-CoV-2, Análise de *clusters*, Algoritmos, Fenótipos, Unidades de terapia intensiva

## Abstract

**Objective:**

To identify more severe COVID-19 presentations.

**Methods:**

Consecutive intensive care unit-admitted patients were subjected to a stepwise clustering method.

**Results:**

Data from 147 patients who were on average 56 ± 16 years old with a Simplified Acute Physiological Score 3 of 72 ± 18, of which 103 (70%) needed mechanical ventilation and 46 (31%) died in the intensive care unit, were analyzed. From the clustering algorithm, two well-defined groups were found based on maximal heart rate [Cluster A: 104 (95%CI 99 - 109) beats per minute *versus* Cluster B: 159 (95%CI 155 - 163) beats per minute], maximal respiratory rate [Cluster A: 33 (95%CI 31 - 35) breaths per minute *versus* Cluster B: 50 (95%CI 47 - 53) breaths per minute], and maximal body temperature [Cluster A: 37.4 (95%CI 37.1 - 37.7)°C *versus* Cluster B: 39.3 (95%CI 39.1 - 39.5)°C] during the intensive care unit stay, as well as the oxygen partial pressure in the blood over the oxygen inspiratory fraction at intensive care unit admission [Cluster A: 116 (95%CI 99 - 133) mmHg *versus* Cluster B: 78 (95%CI 63 - 93) mmHg]. Subphenotypes were distinct in inflammation profiles, organ dysfunction, organ support, intensive care unit length of stay, and intensive care unit mortality (with a ratio of 4.2 between the groups).

**Conclusion:**

Our findings, based on common clinical data, revealed two distinct subphenotypes with different disease courses. These results could help health professionals allocate resources and select patients for testing novel therapies.

## Abstract (pt)

**Objetivo:**

Identificar apresentações mais graves de COVID-19.

**Métodos:**

Pacientes consecutivamente admitidos à unidade de terapia intensiva foram submetidos à análise de *clusters* por meio de método de explorações sequenciais

**Resultados:**

Analisamos os dados de 147 pacientes, com média de idade de 56 ± 16 anos e *Simplified Acute Physiological Score* 3 de 72 ± 18, dos quais 103 (70%) demandaram ventilação mecânica e 46 (31%) morreram na unidade de terapia intensiva. A partir do algoritmo de análise de *clusters*, identificaram-se dois grupos bem definidos, com base na frequência cardíaca máxima [Grupo A: 104 (IC95% 99 - 109) batimentos por minuto *versus* Grupo B: 159 (IC95% 155 - 163) batimentos por minuto], frequência respiratória máxima [Grupo A: 33 (IC95% 31 - 35) respirações por minuto *versus* Grupo B: 50 (IC95% 47 - 53) respirações por minuto] e na temperatura corpórea máxima [Grupo A: 37,4 (IC95% 37,1 - 37,7)ºC *versus* Grupo B: 39,3 (IC95% 39,1 - 39,5)ºC] durante o tempo de permanência na unidade de terapia intensiva, assim como a proporção entre a pressão parcial de oxigênio no sangue e a fração inspirada de oxigênio quando da admissão à unidade de terapia intensiva [Grupo A: 116 (IC95% 99 - 133) mmHg *versus* Grupo B: 78 (IC95% 63 - 93) mmHg]. Os subfenótipos foram distintos em termos de perfis inflamatórios, disfunções orgânicas, terapias de suporte, tempo de permanência na unidade de terapia intensiva e mortalidade na unidade de terapia intensiva (com proporção de 4,2 entre os grupos).

**Conclusão:**

Nossos achados, baseados em dados clínicos universalmente disponíveis, revelaram dois subfenótipos distintos, com diferentes evoluções de doença. Estes resultados podem ajudar os profissionais de saúde na alocação de recursos e seleção de pacientes para teste de novas terapias.

## INTRODUCTION

The severe clinical presentation of 2019 coronavirus disease (COVID-19) requiring admission to the intensive care unit (ICU) is associated with high mortality.^(1)^ Early clinical deterioration is mainly associated with nonpulmonary organ dysfunctions and carries the highest mortality.^(2)^ Moreover, the precocious recognition of more severe forms of the disease is essential.

In acute respiratory distress syndrome (ARDS) patients, clinical, laboratory, and inflammatory data are capable of identifying subphenotypes of more severe presentations^(3-5)^ and, perhaps, guiding respiratory support.^(6)^ COVID-19 patients share some characteristics, predominantly laboratory, which are capable of disclosing the more severe ones.^(7,8)^ Despite the large amount of recent literature published on COVID-19, it is still a new disease, and there is a lack of clinical information about its evolution. Moreover, at bedside, promptness in the ascertainment of information is crucial for making critical decisions.

Therefore, the aim of this study was to identify if there are clinical characteristics, at ICU admission and stay, able to identify the more severe clinical presentations of COVID-19 patients.

## METHODS

This is a retrospective cohort study of critical COVID-19 patients. Data were retrieved from a prospectively collected database from March 19, 2020 to August 3, 2020, which was derived from a single 12-bed ICU at an academic tertiary care center in São Paulo, Brazil. The Research Ethics Committee of *Hospital das Clínicas* of the *Universidade de São Paulo* approved the study protocol (number 107.443), and Informed Consent was waived because of the observational nature of the study.

All patients admitted to the ICU with suspected or confirmed critical COVID-19 were included in this analysis. Patients in whom COVID-19 suspicion was low and *reverse transcription*-*polymerase chain reaction* (RT-PCR) for severe acute respiratory syndrome coronavirus 2 (SARS-CoV-2), serology for SARS-CoV-2 and/or chest tomography were not suggestive of the disease were excluded from the analysis.

### Patient care

In the ICU, patients received organ support according to the current best evidence, without the use of antibiotics (unless coinfection or superinfection was strongly suspected or confirmed)^(9)^ or antiviral drugs (unless in a research protocol).^(10)^ However, prior to ICU transfer, in the emergency setting, most patients did receive at least one dose of antimicrobials, mostly ceftriaxone, azithromycin and/or oseltamivir. Thromboembolism prophylaxis was performed with 40mg of enoxaparin or 15,000 IU of unfractionated heparin.^(11,12)^ Corticosteroids were used as methylprednisolone 1 - 2mg/kg/day for 14 days and tapered up to 28 days.^(13-16)^ Both lung protective mechanical ventilation and prone positioning were used as classically described.^(17,18)^ Driving pressure was used only occasionally to titrate positive end-expiratory pressure (PEEP) in some patients but not as a bedside target variable.^(19)^ Patients were intubated only secondary to severe hypoxemia or severe respiratory distress; thus, no patient was intubated early to avoid self-inflicted lung injury.^(20)^ Neuromuscular blockade was used only in the presence of severe asynchrony or air hunger.^(21)^ The cumulative fluid balance was targeted to zero as soon as possible.^(22)^ Corticosteroids were used in almost all patients.^(16,23,24)^ Because of the extensive human and economic resource burdens, extracorporeal membrane oxygenation (ECMO) was used only in severely hypoxemic patients (when oxygen partial pressure in the blood over the oxygen inspiratory - PaO_2_/FiO_2_ - ratio was persistently lower than 50mmHg despite rescue maneuvers), in patients ventilated up to 7 days, younger than 60 years old, and without severe comorbidities. Extracorporeal membrane oxygenation was not used to treat refractory hypercapnia; instead, high-frequency positive pressure ventilation (HFPPV) was frequently used. These ECMO criteria were not in line with the current literature^(25,26)^ but were adapted to be suitable for more severe disease presentations during the pandemic outbreak.

### Analyzed variables

Clustering analysis was used to characterize and aggregate patients. Furthermore, the variable selection to be clustered was based on clinical simplicity, availability, and low cost. Therefore, we chose to include vital signs, namely, heart rate (HR), respiratory rate (RR), and temperature, all collected every 2 hours during the whole ICU stay. Furthermore, PaO_2_/FiO_2_ at the time of ICU admission was also used for clustering. After clustering, organ dysfunction, organ support, and clinical outcome data were compared between the clusters. The creatinine level was evaluated through the worst value documented variation from baselineto partially adjust the current creatinine value to prior chronic renal impairment.

### Statistical analysis

The quantitative data are presented as the mean ± standard deviation, with the exception of ICU length of stay and days on mechanical ventilation, which are presented as the median [25^th^ percentile and 75^th^ percentile]. The comparisons between survivors and nonsurvivors were performed using a t-test assuming equal variances, the Mann-Whitney test, a chi-squared test or Fisher’s exact test, as appropriate. The aforementioned tetrad of cardinal indicators was the substrate for clustering. These indicators were tested and selected in individual combinations until a visual (graphical) clear separation in different groups of k-means. Standardization using Z scores was adopted to mitigate the scale’s differences bias. The expectation maximization method was applied through the Microsoft clustering algorithm, carried out by Power BI software, in a multistep approach, and the number of clusters (k) was defined by the means of two different systems, automatically by the program’s algorithm, and by the elbow method prediction model. A combinatorial analysis of the four measuring scales was then performed. Given the same dataset, different initial conditions may generate considerably dissimilar clusters,^(25,26)^ which underpins this multifaceted processing. Moreover, a trinomial subanalysis allowed the elaboration of dispersion diagrams, favoring visualization, an intuitive way to perceive and to validate clusters.^(25,26)^ Subsequently, the method’s findings were scrutinized and then merged, with avoidance of superimposed data being assured. The resulting dataset was further refined by preserving only the data points constant in all models to potentiate cluster solidity. On the other hand, the price paid was the shrinking of the sample size. Finally, the cluster’s internal quality was ascertained through reclustering, now taking into account supplementary nonbinary variables, a recounted system for validating results and evaluating group stability.^(25,26)^ The arising groups were compared with the parent clusters, and the matching rate was measured. Confidence intervals (95%) were calculated as usual. R version 4.0.2 free-source software was used for the nonclustering analyses.

## RESULTS

Data from 147 consecutive patients were gathered, of which the data from three patients were excluded after confirmation of alternate diagnoses. Table 1 shows the general characteristics of patients stratified according to survival, where survivors showed substantially lower Simplified Acute Physiological Score 3 (SAPS 3) values. Despite the clinically high suspicion of COVID-19, RT-PCR was positive in only 101 patients (69%). In table 2, organ failure and ICU support are shown; in the survivor’s group, lower maximal SOFA with the exception of the hematological domain, less invasive mechanical ventilation, less neuromuscular blockade, less prone position, less vasopressors, less continuous renal replacement therapy and less antibiotics were needed. The ICU outcomes are shown in table 3; there were 46 nonsurvivors (31%).

**Table 1:** General patient characteristics of the entire group of patients stratified according to survival outcome

Characteristics	Whole group	Survivors	Nonsurvivors	p value*
	n = 147	n = 101	n = 46	
Age (years)	56 ± 15	54 ± 15	62 ± 14	0.002
Male gender	86 (59)	56 (55)	30 (65)	0.350
SAPS 3	72 ± 18	67 ± 18	82 ± 15	< 0.001
ECOG	1.42 ± 1.16	1.35 ± 1.19	1.57 ± 1.07	0.297
ABG at admission				
PaO_2_/FiO_2_ (mmHg)	128 ± 94	146 ± 105	93 ± 54	0.002
PaO_2_/FiO_2_ categories				0.006
; 100mmHg	62 (42)	32 (32)	30 (65)	
100 to < 200mmHg	51 (35)	39 (39)	12 (26)	
200 to < 300mmHg	14 (10)	11 (11)	3 (7)	
≥ 300 mmHg	6 (4)	6 (6)	0 (0)	
Lactate (mmol/L)	2.28 ± 2.66	1.70 ± 1.11	3.42 ± 4.10	< 0.001
pH	7.36 ± 0.11	7.39 ± 0.08	7.31 ± 0.13	< 0.001
PaCO_2_ (mmHg)	44 ± 13	41 ± 10	50 ± 16	< 0.001
SBE (mEq/L)	- 1.17 ± 4.63	- 0.28 ± 3.88	- 2.91 ± 5.45	0.002
Patient source				0.049
Another ICU	56 (38)	35 (35)	21 (46)	
Emergency room	55 (37)	34 (34)	21 (46)	
Ward	31 (21)	27 (27)	4 (9)	
Operating room	5 (3)	5 (5)	0 (0)	
Causes of ICU admission				0.585
Respiratory failure	122 (83)	81 (80)	41 (89)	
Sepsis/septic shock	14 (10)	9 (9)	5 (11)	
Cardiogenic shock	2 (1)	2 (2)	0 (0)	
Neurologic syndromes	4 (3)	4 (4)	0 (0)	
Acute heart failure	2 (1)	2 (2)	0 (0)	
Acute renal failure	2 (1)	2 (2)	0 (0)	
High-risk postoperative	1 (1)	1 (1)	0 (0)	
Comorbidities				
Hypertension	88 (60)	54 (53)	34 (74)	0.030
Heart failure	23 (16)	17 (17)	6 (13)	0.733
Diabetes	43 (29)	27 (27)	16 (35)	0.424
Neoplasm	16 (11)	10 (10)	6 (13)	0.778
Smoking	15 (10)	8 (8)	7 (15)	0.289
Chronic renal failure	11 (7)	9 (9)	2 (4)	0.524
Stroke	2 (1)	2 (2)	0 (0)	0.847
COPD	6 (4)	2 (2)	4 (9)	0.145
AIDS	3 (2)	3 (3)	0 (0)	0.581

SAPS 3 - Simplified Acute Physiological Score 3; ECOG - Eastern Cooperative Oncology Group; ABG - arterial blood gas; PaO2/FiO2 - oxygen partial pressure in the blood over the oxygen inspiratory fraction; PaCO2 - partial pressure of carbon dioxide in arterial blood; SBE - standard base excess; ICU - intensive care unit; COPD - chronic obstructive pulmonary disease.

* These p-values result from comparisons between survivors and nonsurvivors. Results expressed as mean ± standard deviation or n (%).

**Table 2:** Organ dysfunctions and support of the entire group of patients stratified according to survival outcome

Characteristics	Whole group	Survivors	Nonsurvivors	p value*
n = 147	n = 101	n = 46
Maximal SOFA during the ICU stay†				
Respiratory	3.16 ± 1.03	2.87 ± 1.07	3.81 ± 0.50	< 0.001
Cardiovascular	2.28 ± 1.81	1.72 ± 1.78	3.56 ± 1.12	< 0.001
Renal	2.26 ± 1.70	1.70 ± 1.63	3.51 ± 1.05	< 0.001
Neurological	2.48 ± 1.68	1.89 ± 1.66	3.81 ± 0.70	< 0.001
Hepatic	0.45 ± 0.89	0.21 ± 0.54	1.00 ± 1.23	< 0.001
Hematological	0.29 ± 0.68	0.23 ± 0.64	0.44 ± 0.73	0.086
Respiratory support				
Mechanical ventilation	103 (70)	61 (60)	42 (91)	< 0.001
Noninvasive mechanical ventilation	55 (37)	40 (40)	15 (33)	0.529
Neuromuscular blockade	55 (37)	22 (22)	33 (72)	< 0.001
High-flow nasal cannula	31 (21)	22 (22)	9 (20)	0.930
Prone position	26 (18)	13 (13)	13 (28)	0.042
Inhaled nitric oxide	7 (5)	3 (3)	4 (9)	0.274
ECMO	5 (3)	1 (1)	4 (9)	0.058
Nonrespiratory support				
Palliative care < 48 hours‡	23 (16)	6 (6)	17 (37)	< 0.001
Vasopressors	84 (57)	43 (43)	41 (89)	< 0.001
Inotropes	9 (6)	5 (5)	4 (9)	0.612
Slow low-efficiency dialysis	21 (14)	13 (13)	8 (17)	0.637
Continuous renal replacement therapy	17 (12)	5 (5)	12 (26)	0.001
Antibiotics§	30 (20)	10 (10)	20 (43)	< 0.001
Vital signs and glycemia during ICU stay				
Maximal heart rate (beats/minute)	131 ± 23	127 ± 23	141 ± 21	0.001
Minimal mean arterial pressure (mmHg)	56 ± 19	58 ± 20	51 ± 16	0.036
Maximal respiratory rate (breaths/minute)	46 ± 13	44 ± 13	50 ± 10	0.005
Minimal peripheral oxygen saturation (%)	76 ± 14	78 ± 14	73 ± 11	0.021
Maximal body temperature (°C)	38.31 ± 0.90	38.14 ± 0.85	38.68 ± 0.91	0.001
Minimal glycemia (mg/dL)	68 ± 28	68 ± 24	67 ± 36	0.834
Maximal glycemia (mg/dL)	242 ± 131	211 ± 109	308 ± 151	< 0.001
Laboratory data¶				
Maximal plasma D-dimer (ng/mL)	14.271 ± 28.588	8.310 ± 18.539	26.017 ± 39.686	0.003
Maximal plasma LDH (U/L)	640 ± 690	502 ± 275	925 ± 1095	0.003
Minimal lymphocytes (cells/mm3)	714 ± 459	822 ± 507	507 ± 246	< 0.001

SOFA - Sequential Organ Failure Assessment; ICU - intensive care unit; ECMO - extracorporeal membrane oxygenation; LDH - lactate dehydrogenase.

* These p values result from comparisons between survivors and nonsurvivors; † these values are the maximal Sequential Organ Failure Assessment extracted daily from each dimension of the Sequential Organ Failure Assessment; ‡ these are the patients on exclusive palliative care within the first 48 hours of intensive care unit stay; § these numbers include all the antibiotics used during the intensive care unit stay for coinfections or superinfections; ¶ these laboratory data were obtained at any time during the intensive care unit stay. Results expressed as mean ± standard deviation, n (%).

**Table 3:** Intensive care unit outcomes of the entire group of patients stratified according to survival outcome

Characteristics	Whole group	Survivors	Nonsurvivors	p value
	n = 147	n = 101	n = 46	
ICU length-of-stay (days)	7 [3 - 13]	6 [3 - 12]	9 [5 - 14]	0.072
Days on invasive mechanical ventilation	5 [3 - 9]	4 [0 - 7]	7 [3 - 11]	0.009
ICU mortality	46 (31)	---	---	---

ICU - intensive care unit. * These p-values result from comparisons between survivors and nonsurvivors. Results expressed as the median [25th percentile - 75th percentile] or n (%).

**Figure 1:**
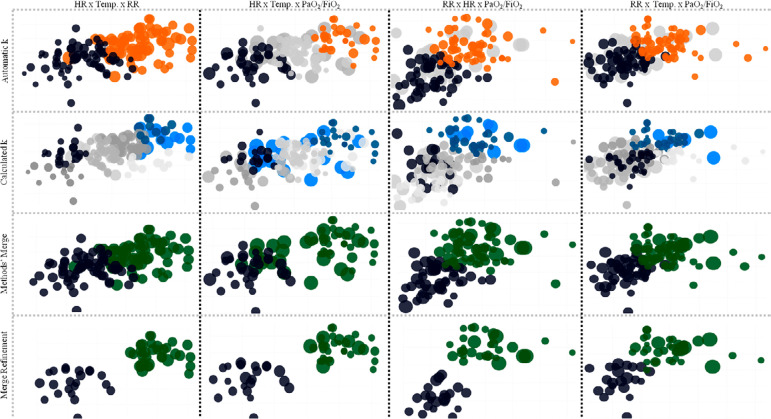
Graphical representation of stepwise clustering. k - minimized through the squared Euclidean distances within clusters. HR - heart rate; Temp - body temperature; PaO2/FiO2 - oxygen partial pressure in the blood over the oxygen inspiratory fraction; RR - respiratory rate. The merge refinement represents the probabilistic distribution of the clusters according to the expectation maximization algorithm.

The clustering process led to two well-defined assemblies (Figure 1 and Table 4), hereinafter denominated Cluster A (n = 22) and Cluster B (n = 35), which had comparable demographic features but contrasting clinical and laboratory variables. There were five patients in each cluster with missing data for the plasma D-dimer level and three and six patients with missing values for CRP in Clusters A and B, respectively. Foremost, there were disparities in the parameters that shaped the clusters *per se*. The minimal admission PaO_2_/FiO_2_ ratio was lower in cluster B [Cluster A: 116 (95%CI 99 - 133) mmHg *versus* 78 (95%CI 63 - 93) mmHg], as well as the maximal RR [Cluster A: 33 (95%CI 31 - 35) breaths per minute *versus* Cluster B: 50 (95%CI 47 - 53) breaths per minute], maximal HR [Cluster A: 104 (95%CI 99 - 109) beats per minute *versus* Cluster B: 159 (95%CI 155 - 163) beats per minute] and temperature [Cluster A: 37.4 (95%CI 37.1 - 37.7)°C *versus* Cluster B: 39.3 (95%CI 39.1 - 39.5)°C] were higher during the ICU stay. All the respiratory, cardiovascular and renal support metrics differed between the groups, both in frequency and duration, with an increased intervention need in cluster B. The white cell counts in Cluster B were appreciably increased when set against the findings of Cluster A, as were the CRP levels. Thrombotic events occurred more often in Cluster B, and the maximal plasma D-dimer levels was also higher in this cluster. Finally, the SAPS 3 and maximal Sequential Organ Failure Assessment - SOFA (in all six domains) score differences surfaced in the cluster comparison, which reinforced a highly relevant mortality rate variance that was 4·2 times higher in Cluster B that that in Cluster A.

**Table 4:** Characteristics of the clusters

Characteristics	Cluster A	Cluster B	p value*
	n = 22/ n = 10	n = 35/n = 30	
Age (years)	58 ± 16/58 ± 15	55 ± 17/56 ± 15	0.461/0.226
Male gender	15 (68)/7 (70)	23 (66)/19 (63)	0.923/0.702
SAPS3	65 ± 17/65 ± 17	82 ± 16/81 ± 15	< 0.001/0.275
ECOG	1.68 ± 1.36/1.68 ± 1.35	1.15 ± 1.08/1.03 ± 1.02	0.108/0.370
Comorbidities			
Hypertension	13 (59)/7 (70)	21 (60)/18 (60)	0.834/0.850
Diabetes	5 (23)/2 (20)	13 (37)/11 (37)	0.397/0.559
Obesity	3 (14)/3 (30)	7 (20)/6 (20)	0.797/0.827
Heart failure	5 (23)/1 (10)	4 (11)/3 (10)	0.444/1.000
COPD/asthma	2 (9)/1 (10)	3 (9)/2 (7)	0.679/0.729
Smoking	1 (5)/1 (10)	2 (6)/2 (7)	0.677/0.729
Neoplasm	4 (18)/3 (30)	2 (6)/1 (3)	0.294/0.068
Chronic renal failure	0 (0)	2 (6)/2 (7)	0.688/0.402
Immunosuppression	0 (0)	3 (9)/3 (10)	0.423/0.729
Respiratory support			
High-flow nasal cannula (n° of patients); (days)	2 (9);0 [0 - 0]	9 (26); 0 [0 - 1]	0.229; 0.058
	1 (10);0 [0 - 0]	8 (27); 0 [0 - 1.5]	0.512; 0.194
Noninvasive mechanical ventilation (n° of patients); (days)	5 (23);0 [0 - 0]	18 (51); 1 [0 - 4]	0.061; 0.014
	3 (30);0 [0 - 1.5]	16 (53); 1 [0 - 4]	0.361; 0.206
Mechanical ventilation (n° of patients); (days)	8 (36);0 [0 - 2]	35 (100); 7 [5 - 13.5]	< 0.001; < 0.001
	4 (40);0 [0 - 1.8]	30 (100); 8 [5 - 13.3]	0.002; < 0.001
Need for reintubation (n° of patients); (occurrences)	0 (0); 0	18 (51); 0.66 ± 0.76	< 0.001; < 0.001
		16 (53); 0.63 ± 0.67	0.009; 0.005
Neuromuscular blockade (n° of patients); (days)	4 (18);0.18 ± 0.39	26 (74); 1.89 ± 2.29	< 0.001; 0.001
	4 (40);0.40 ± 0.52	23 (77); 2.10 ± 2.40	0.079; 0.033
Prone position (n° of patients); (days)	0 (0); 0	15 (43); 1.00 ± 1.39	0.001; 0.001
		14 (47); 1.10 ± 1.45	0.022; 0.022
Inhaled nitric oxide (n° of patients); (days)	0 (0); 0	5 (14); 0.34 ± 0.94	0.169; 0.093
		4 (13); 0.37 ± 1.00	0.543; 0.257
ECMO (n° of patients); (days)	1 (5); 0.05 ± 0.21	2 (6); 0.43 ± 2.08	0.677; 0.394
	1 (10); 0.10 ± 0.32	1 (3); 0.40 ± 2.19	0.402; 0.671
Nonrespiratory support			
Vasopressors (n° of patients); (days)	6 (27); 0.59 ± 1.37	31 (89); 2.77 ± 2.18	< 0.001; < 0.001
	1 (10); 1.00 ± 1.89	26 (87); 2.80 ± 2.31	< 0.001; 0.032
Renal replacement therapy (n° of patients); (days)	1 (5); 0.05 ± 0.21	16 (46); 1.23 ± 2.02	0.003; 0.008
	1 (5); 0.10 ± 0.32	15 (50); 1.37 ± 2.13	0.062; 0.070
Antibiotics†	18 (82)/8 (80)	32 (91)/27 (90)	0.508/0.783
ABG oxygenation values			
PaO_2_/FiO_2_ (mmHg)	116 ± 40/114 ± 41	78 ± 44/78 ± 45	0.002/0.034
PaO_2_/FiO_2_ categories			
< 100mmHg	8 (36)/4 (40)	28 (80)/25 (83)	0.002/0.025
100 to < 200mmHg	13 (59)/6 (60)	6 (17)/4 (13)	0.003/0.011
200 to < 300mmHg	1 (5)/0 (0)	1 (3)/1 (3)	0.688/0.559
≥ 300mmHg	0 (0)	0 (0)	
Vital signs during the ICU stay			
Maximal heart rate (beats/minute)	104 ± 13/105 ± 12	159 ± 11/159 ± 11	< 0.001/< 0.001
Maximal respiratory rate (breaths/minute)	33 ± 5/35 ± 5	50 ± 10/49 ± 8	< 0.001/< 0.001
Maximal body temperature (°C)	37.4 ± 0.8/37.7 ± 1.0	39.3 ± 0.6/39.3 ± 0.7	< 0.001/< 0.001
Laboratory data‡			
Maximal white cell count (cells/mm3)	13.906 ± 8.089/15.883 ± 9.765	25.788 ± 10.828/25.701 ± 11.007	< 0.001/0.017
Maximal C-reactive protein (mg/L)	147 ± 123/163 ± 161	245 ± 154/257 ± 158	0.025/0.160
Maximal plasma D-dimer (ng/mL)	8.833 ± 15.953/13.976 ± 21.925	25.408 ± 40.260/27.608 ± 42.590	0.112/0.394
Creatinine variation	0.74 ± 1.10/1.07 ± 1.54	3.63 ± 2.46/3.66 ± 2.41	< 0.001/0.003
Maximal SOFA score during the ICU stay§			
Respiratory	2.73 ± 1.12/3.40 ± 0.84	3.89 ± 0.32/3.90 ± 0.31	< 0.001/0.008
Cardiovascular	0.86 ± 1.55/1.20 ± 1.75	3.60 ± 1.14/3.57 ± 1.22	< 0.001/< 0.001
Renal	1.05 ± 1.68/1.40 ± 1.84	3.26 ± 1.15/3.27 ± 1.20	< 0.001/< 0.001
Neurological	1.14 ± 1.42/1.50 ± 1.65	3.83 ± 0.62/3.83 ± 0.65	< 0.001/< 0.001
Hepatic	0.23 ± 0.61/0.40 ± 0.84	0.94 ± 1.03/0.93 ± 1.05	0.005/0.154
Hematological	0.27 ± 0.77/0.50 ± 1.08	0.49 ± 0.82/0.50 ± 0.86	0.331/1.000
Thrombotic event	4 (18)/2 (20)	10 (29)/10 (33)	0.568/0.690
ICU length-of-stay - days	2 [1.25 - 3.75]/6 [2 - 6]	13 [8 - 21]/15 [8 - 22]	< 0.001/0.006
ICU mortality	3 (14)/3 (30)	20 (57)/16 (53)	0.003/0.361

SAPS 3 - Simplified Acute Physiological Score 3; ECOG - Eastern Cooperative Oncology Group score; COPD - chronic obstructive pulmonary disease; ECMO - extracorporeal membrane oxygenation; PaO_2_/FiO_2_ - oxygen partial pressure in the blood over the oxygen inspiratory fraction; ICU - intensive care unit; SOFA - Sequential Organ Failure Assessment. Data in gray reflect findings in the subset of patients with reverse transcription-polymerase chain reaction-confirmed COVID-19.

* These p values result from comparisons between survivors and nonsurvivors; † these numbers accomplish all the antibiotics used during the intensive care unit stay for coinfections or superinfections; ‡ these laboratory data were obtained at any time of the intensive care unit stay; § these values are the maximal Sequential Organ Failure

**Figure 2:**
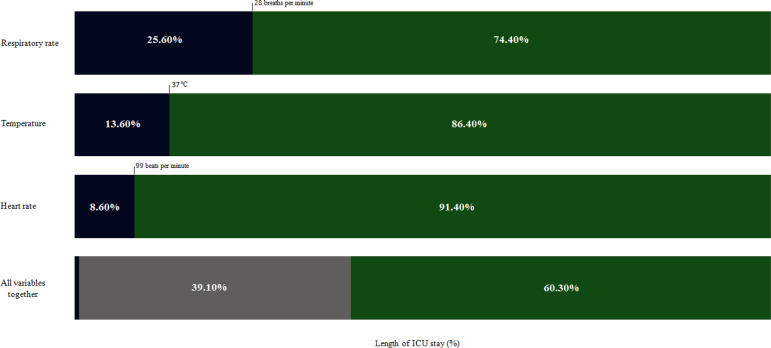
The time length of the intensive care unit stay with the respiratory rate, heart rate, temperature and all variables together compatible with each cluster. ICU - intensive care unit. Blue represents the percentage of the time of the intensive care unit stay compatible with Cluster A. Green represents the percentage of the time of the intensive care unit stay compatible with Cluster B. Gray represents the percentage of the time of the intensive care unit stay, where there were variables in ranges compatible with both clusters.

The daily mean variation amplitude was wide for the three physiological parameters. In Cluster A, the HR average oscillation was 72 - 99 beats per minute, the RR was 16 - 28 breaths per minute, and the temperature was 35.5 - 37.0°C. In Cluster B, the observed fluctuations were 92 - 126 beats per minute, 19 - 36 breaths per minute, and 35.8 - 38.9°C, respectively. Assuming the upper limits of the range as the boundary of Cluster A, considering the whole group of patients, only in 8.6% of the observed time the HR was compatible with the Cluster A subphenotype. The same occurred in 25.6% and 13.6% of the observed time for RR and temperature, respectively (Figure 2). The parameter interrelationships were also heterogeneous. The three variables stood together consistently compatible with Cluster B in 60.3% of the observed time, and only in 0.6% of the observed time were the three variables together compatible with Cluster A (Figure 2).

## DISCUSSION

Considering only ICU COVID-19 patients, heterogeneity remains a marked feature. In our patients, there were several clinical-laboratory differences in regard to general characteristics, organ failure, and organ support between severe COVID-19 patients who survived and those who did not survive their ICU stay. However, simple clinical variables such as HR, RR, and body temperature during the ICU stay and the PaO_2_/FiO_2_ ratio at the time of ICU admission were able to separate the COVID-19 patients into two different subphenotypes.

Some patient characteristics were different between the survivors and nonsurvivors at ICU admission, such as the SAPS 3 score, age, PaO_2_/FiO_2_ ratio, lactate and acid-base status, all of which are in line with the current literature.^(27-29)^

Models for the prediction of unfavorable evolution of COVID-19 have been proposed. There are different outcome prediction models, taking into account demographic data,^(2)^ laboratory data,^(2)^ and the combination of clinical plus radiologic features.^(2)^ Otherwise, no study has been dedicated to exploring only bedside clinical data. In this way, also based upon the premise of different courses of disease, the clustering of COVID-19 in subphenotypes has been reported. The approach adopted by Azoulay et al.^(2)^ included clinical and laboratory multiparametric analyses, eliciting findings consistent with risk-prediction studies. The refinement of the clustering method resulted in a reduced sample size in both clusters; moreover, this technique reduces the sensitivity of cluster characteristics, otherwise enhancing their specificity.^(30)^

It is interesting to note that the time spent with vital signs within the range of Cluster A was low, probably because those patients had a shorter ICU stay. Moreover, this physiological behavior brings a consistent clinical meaning of a good outcome pool of patients.

The purpose of the present study, which used predominantly clinical data, was to offer a cost-effective alternative for resource allocation guidance that eventually may aid in the selection of candidates for testing novel therapies or even for the early implementation of treatments in the future.

The limitations of our study include the sample size, the single-center source of the patients, the subjectivity that permeated the selection of variables for clustering, and the lack of validation in an external cohort. In compensation, our proposition was built in such a way that the wide heterogeneity of resource availability across centers would not become a constraint to prospective studies in different or larger populations. Furthermore, since patient stratification is a critical task in clinical decision making, bedside guiding elements could thus facilitate and hasten these judgements. An additional strength of the study is the considerable premorbid similarity between the individuals from both groups, which minimized the confounding factors. Additionally, the academic tertiary health service status, together with Brazil’s (and São Paulo’s) broad sociocultural diversity, may have contributed to reducing the underrepresentation of population subsets.

## CONCLUSION

This study was able to identify two clinically distinct subphenotypes of COVID-19 patients in accordance with disease severity. Maximal heart rate, body temperature, respiratory rate and the intensive care unit admission oxygen partial pressure in the blood over the oxygen inspiratory ratio are bedside variables that can help identify more severe COVID-19 patients.

## References

[1] Rieg S, von Cube M, Kalbhenn J, Utzolino S, Pernice K, Bechet L, Baur J, Lang CN, Wagner D, Wolkewitz M, Kern WV, Biever P, COVID UKF Study Group (2020). COVID-19 in-hospital mortality and mode of death in a dynamic and non-restricted tertiary care model in Germany. PLoS One.

[2] Azoulay E, Fartoukh M, Darmon M, Géri G, Voiriot G, Dupont T (2020). Increased mortality in patients with severe SARS-CoV-2 infection admitted within seven days of disease onset. Intensive Care Med.

[3] Kitsios GD, Yang L, Manatakis DV, Nouraie M, Evankovich J, Bain W (2019). Host-response subphenotypes offer prognostic enrichment in patients with or at risk for acute respiratory distress syndrome. Crit Care Med.

[4] Spadaro S, Park M, Turrini C, Tunstall T, Thwaites R, Mauri T (2019). Biomarkers for acute respiratory distress syndrome and prospects for personalised medicine. J Inflamm (Lond).

[5] Calfee CS, Delucchi K, Parsons PE, Thompson BT, Ware LB, Matthay MA, NHLBI ARDS Network (2014). Subphenotypes in acute respiratory distress syndrome: latent class analysis of data from two randomised controlled trials. Lancet Respir Med.

[6] Sinha P, Delucchi KL, McAuley DF, O'Kane CM, Matthay MA, Calfee CS (2020). Development and validation of parsimonious algorithms to classify acute respiratory distress syndrome phenotypes: a secondary analysis of randomised controlled trials. Lancet Respir Med.

[7] Azoulay E, Zafrani L, Mirouse A, Lengliné E, Darmon M, Chevret S (2020). Clinical phenotypes of critically ill COVID-19 patients. Intensive Care Med.

[8] Lin SH, Zhao YS, Zhou DX, Zhou FC, Xu F (2020). Coronavirus disease 2019 (COVID-19): cytokine storms, hyper-inflammatory phenotypes, and acute respiratory distress syndrome. Genes Dis.

[9] Furtado RH, Berwanger O, Fonseca HA, Corrêa TD, Ferraz LR, Lapa MG, Zampieri FG, Veiga VC, Azevedo LCP, Rosa RG, Lopes RD, Avezum A, Manoel ALO, Piza FMT, Martins PA, Lisboa TC, Pereira AJ, Olivato GB, Dantas VCS, Milan EP, Gebara OCE, Amazonas RB, Oliveira MB, Soares RVP, Moia DDF, Piano LPA, Castilho K, Momesso RGRAP, Schettino GPP, Rizzo LV, Neto AS, Machado FR, Cavalcanti AB, COALITION COVID-19 Brazil II Investigators (2020). Azithromycin in addition to standard of care versus standard of care alone in the treatment of patients admitted to the hospital with severe COVID-19 in Brazil (COALITION II): a randomised clinical trial. Lancet.

[10] Cavalcanti AB, Zampieri FG, Rosa RG, Azevedo LC, Veiga VC, Avezum A, Damiani LP, Marcadenti A, Kawano-Dourado L, Lisboa T, Junqueira DLM, de Barros E Silva PGM, Tramujas L, Abreu-Silva EO, Laranjeira LN, Soares AT, Echenique LS, Pereira AJ, Freitas FGR, Gebara OCE, Dantas VCS, Furtado RHM, Milan EP, Golin NA, Cardoso FF, Maia IS, Hoffmann Filho CR, Kormann APM, Amazonas RB, Bocchi de Oliveira MF, Serpa-Neto A, Falavigna M, Lopes RD, Machado FR, Berwanger O, Coalition Covid-19 Brazil I Investigators (2020). Hydroxychloroquine with or without Azithromycin in Mild-to-Moderate Covid-19. N Engl J Med.

[11] Schünemann HJ, Cushman M, Burnett AE, Kahn SR, Beyer-Westendorf J, Spencer FA (2018). American Society of Hematology 2018 guidelines for management of venous thromboembolism: prophylaxis for hospitalized and nonhospitalized medical patients. Blood Adv.

[12] Samama MM, Cohen AT, Darmon JY, Desjardins L, Eldor A, Janbon C, Prophylaxis in Medical Patients with Enoxaparin Study Group (1999). A comparison of enoxaparin with placebo for the prevention of venous thromboembolism in acutely ill medical patients. N Engl J Med.

[13] Meduri GU, Headley AS, Golden E, Carson SJ, Umberger RA, Kelso T (1998). Effect of prolonged methylprednisolone therapy in unresolving acute respiratory distress syndrome: a randomized controlled trial. JAMA.

[14] Meduri GU, Golden E, Freire AX, Taylor E, Zaman M, Carson SJ (2007). Methylprednisolone infusion in early severe ARDS: results of a randomized controlled trial. Chest.

[15] Steinberg KP, Hudson LD, Goodman RB, Hough CL, Lanken PN, Hyzy R, Thompson BT, Ancukiewicz M, National Heart, Lung, and Blood Institute Acute Respiratory Distress Syndrome (ARDS) Clinical Trials Network (2006). Efficacy and safety of corticosteroids for persistent acute respiratory distress syndrome. N Engl J Med.

[16] Sterne JA, Murthy S, Diaz JV, Slutsky AS, Villar J, Angus DC, WHO Rapid Evidence Appraisal for COVID-19 Therapies (REACT) Working Group (2020). Association between administration of systemic corticosteroids and mortality among critically ill patients with COVID-19: a meta-analysis. JAMA.

[17] Brower RG, Matthay MA, Morris A, Schoenfeld D, Thompson BT, Wheeler A, Acute Respiratory Distress Syndrome Network (2000). Ventilation with lower tidal volumes as compared with traditional tidal volumes for acute lung injury and the acute respiratory distress syndrome. N Engl J Med.

[18] Guérin C, Reignier J, Richard JC, Beuret P, Gacouin A, Boulain T, Mercier E, Badet M, Mercat A, Baudin O, Clavel M, Chatellier D, Jaber S, Rosselli S, Mancebo J, Sirodot M, Hilbert G, Bengler C, Richecoeur J, Gainnier M, Bayle F, Bourdin G, Leray V, Girard R, Baboi L, Ayzac L, PROSEVA Study Group (2013). Prone positioning in severe acute respiratory distress syndrome. N Engl J Med.

[19] Sahetya SK, Hager DN, Stephens RS, Needham DM, Brower RG (2020). PEEP titration to minimize driving pressure in subjects with ARDS: a prospective physiological study. Respir Care.

[20] Tobin MJ, Laghi F, Jubran A (2020). P-SILI is not justification for intubation of COVID-19 patients. Ann Intensive Care.

[21] Moss M, Huang DT, Brower RG, Ferguson ND, Ginde AA, Gong MN (2019). Early neuromuscular blockade in the acute respiratory distress syndrome. N Engl J Med.

[22] Wiedemann HP, Wheeler AP, Bernard GR, Thompson BT, Hayden D, deBoisblanc B, National Heart, Lung, and Blood Institute Acute Respiratory Distress Syndrome (ARDS) Clinical Trials Network (2006). Comparison of two fluid-management strategies in acute lung injury. N Engl J Med.

[23] Horby P, Lim WS, Emberson JR, Mafham M, Bell JL, Linsell L, RECOVERY Collaborative Group (2021). Dexamethasone in hospitalized patients with Covid-19. N Engl J Med.

[24] Tomazini BM, Maia IS, Cavalcanti AB, Berwanger O, Rosa RG, Veiga VC, Avezum A, Lopes RD, Bueno FR, Silva MV, Baldassare FP, Costa EL, Moura RA, Honorato MO, Costa AN, Damiani LP, Lisboa T, Kawano-Dourado L, Zampieri FG, Olivato GB, Righy C, Amendola CP, Roepke RM, Freitas DH, Forte DN, Freitas FG, Fernandes CC, Melro LM, Junior GF, Morais DC, Zung S, Machado FR, Azevedo LC, COALITION COVID-19 Brazil III Investigators (2020). Effect of dexamethasone on days alive and ventilator-free in patients with moderate or severe acute respiratory distress syndrome and COVID-19: The CoDEX Randomized Clinical Trial. JAMA.

[25] Combes A, Hajage D, Capellier G, Demoule A, Lavoué S, Guervilly C, Da Silva D, Zafrani L, Tirot P, Veber B, Maury E, Levy B, Cohen Y, Richard C, Kalfon P, Bouadma L, Mehdaoui H, Beduneau G, Lebreton G, Brochard L, Ferguson ND, Fan E, Slutsky AS, Brodie D, Mercat A, EOLIA Trial Group, REVA, ECMONet (2018). Extracorporeal membrane oxygenation for severe acute respiratory distress syndrome. N Engl J Med.

[26] Zampieri FG, Mendes PV, Ranzani OT, Taniguchi LU, Pontes Azevedo LC, Vieira Costa EL (2013). Extracorporeal membrane oxygenation for severe respiratory failure in adult patients: a systematic review and meta-analysis of current evidence. J Crit Care.

[27] Moreno RP, Metnitz PG, Almeida E, Jordan B, Bauer P, Campos RA, Iapichino G, Edbrooke D, Capuzzo M, Le Gall JR, SAPS 3 Investigators (2005). SAPS 3--From evaluation of the patient to evaluation of the intensive care unit. Part 2: Development of a prognostic model for hospital mortality at ICU admission. Intensive Care Med.

[28] Azevedo LC, Park M, Salluh JI, Rea-Neto A, Souza-Dantas VC, Varaschin P, Oliveira MC, Tierno PF, Dal-Pizzol F, Silva UV, Knibel M, Nassar AP Jr, Alves RA, Ferreira JC, Teixeira C, Rezende V, Martinez A, Luciano PM, Schettino G, Soares M, ERICC (Epidemiology of Respiratory Insufficiency in Critical Care) investigators (2013). Clinical outcomes of patients requiring ventilatory support in Brazilian intensive care units: a multicenter, prospective, cohort study. Crit Care.

[29] Maciel AT, Park M (2009). Differences in acid-base behavior between intensive care unit survivors and nonsurvivors using both a physicochemical and a standard base excess approach: a prospective, observational study. J Crit Care.

[30] Kern M, Lex A, Gehlenborg N, Johnson CR (2017). Interactive visual exploration and refinement of cluster assignments. BMC Bioinformatics.

